# Survival from cancer of the oesophagus in England and Wales up to 2001

**DOI:** 10.1038/sj.bjc.6604572

**Published:** 2008-09-23

**Authors:** E Mitry, B Rachet, M J Quinn, N Cooper, M P Coleman

**Affiliations:** 1 Département d'Hépatogastroentérologie et Oncologie Digestive, Centre Hospitalo-Universitaire Ambroise-Paré, 9 avenue Charles de Gaulle, Boulogne F-92100, France; 2 Cancer Research UK Cancer Survival Group, Non-Communicable Disease Epidemiology Unit, Department of Epidemiology and Population Health, London School of Hygiene and Tropical Medicine, Keppel Street, London WC1E 7HT, UK; 3 Social and Health Analysis and Reporting Division, Office for National Statistics (Room FG/114), 1 Myddelton Street, London EC1R 1UW, UK

Oesophageal cancer is the eighth most common cancer in the world, with striking geographical variations in incidence (Parkin, 2001). It accounts for approximately 1 in 40 of all cancers among adults in England and Wales, with an average of some 6000 new cases a year, of which some 60% occur in men (Quinn *et al*, 2001). Incidence has increased steadily in both sexes since the 1970s. Lifetime risks up to the age of 74 years are currently approximately 1% in men and 0.4% in women (Parkin *et al*, 2002). Trends in mortality are similar to those for incidence, because survival has been poor for many years. The incidence of adenocarcinoma of the lower third of the oesophagus and the gastric cardia has been increasing for 20 years in England (Powell and McConkey, 1990; Newnham *et al*, 2003) and in other countries (Bollschweiler *et al*, 2001). More than 90% of squamous cell carcinomas can be attributed to alcohol and tobacco in Europe and North America, whereas the main risk factor for adenocarcinoma is Barrett's oesophagus. Oesophageal cancer has an extremely poor prognosis: the average 5-year relative survival rate among adults diagnosed in 22 European countries during the early 1990s was approximately 10% (Sant *et al*, 2003).

We analysed data for 65 591 patients diagnosed with oesophageal cancer in England and Wales during the period 1986–1999, approximately 84% of those eligible, with follow-up to the end of 2001. Exclusions from analysis were mainly of patients whose recorded survival was zero (10.8%) or whose cancer of the oesophagus was not their first primary malignancy (3.6%). A third (34%) of tumours were squamous carcinomas, but the proportion classified as adenocarcinoma rose from 32 to 44% during the 1990s, continuing a steady increase from 15% in the early 1970s (Coleman *et al*, 1999). Annual incidence rates increased by about one-third in men and women during the period 1986–1999. Trends were similar for all deprivation groups in both sexes, and incidence was lowest in the most affluent groups throughout (Figure 1).

## Survival trends

**Figure 2:**
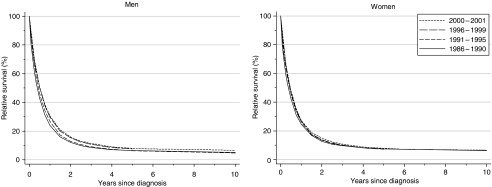
Relative survival (%) up to 10 years after diagnosis by sex and calendar period of diagnosis: England and Wales, adults (15–99 years) diagnosed during 1986–1999 and followed up to 2001. Survival estimated with cohort or complete approach (1986–1990, 1991–1995, 1996–1999) or hybrid approach (2000–2001) (see Rachet *et al*, 2008).

Relative survival at 1 year increased significantly from the late 1980s to the late 1990s in both sexes, from 23.8 to 29.6% in men (fitted, deprivation-adjusted average increase 3.7% every 5 years) and from 24.4 to 26.8% in women (+1.4% every 5 years) (Table 1, Figure 2). Five-year survival increased from 6.3 to 7.6% in men over the same period (average increase +1.7% every 5 years), but there was no change in 5-year survival for women: 7.4% for those diagnosed during 1986–1990 and 7.2% for those diagnosed during 1996–1999. Short-term predictions of survival for patients diagnosed during 2000–2001, using hybrid analysis (Brenner and Rachet, 2004), suggest a small continuing increase in survival at 1, 5 and 10 years after diagnosis (Table 1).

## Deprivation

**Figure 3:**
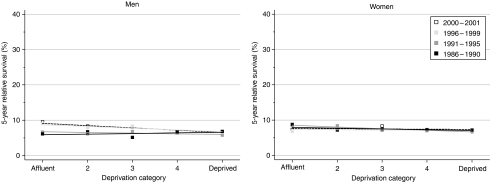
Trends in the deprivation gap in 5-year relative survival (%) by sex and calendar period of diagnosis: England and Wales, adults (15–99 years) diagnosed during 1986–1999 and followed up to 2001.

The deprivation gap was more marked in men (Table 2, Figure 3). For 1-year survival, the deprivation gap remained unchanged at approximately −5% (lower for the most deprived group than for the most affluent), but for survival at 5 and 10 years, the deprivation gap widened significantly every 5 years by −1.4% for 5-year survival and by −2.7% for 10-year survival. For women, the deprivation gap in survival was less marked, and did not change significantly over time.

## Comment

Survival from oesophageal cancer in England and Wales remains very poor, with 5-year relative survival approximately 7.5%, and no major improvement for patients diagnosed between 1986 and 1999. Five-year survival was already 6–7% for patients diagnosed in 1971–1975 (Coleman *et al*, 1999). This suggests a lack of substantial progress in diagnostic or therapeutic management over the last 30 years. However, the slight improvement in 1-year survival for both sexes suggests a decrease in postoperative mortality (Faivre *et al*, 1998). There was a trend towards higher 5-year survival for men, accompanied by a significant increase in the deprivation gap; this was not seen for women. As stage at diagnosis is the main prognostic factor for oesophageal cancer, and incidence trends were similar in all deprivation groups, these trends in 5-year survival may suggest a trend towards earlier stage at diagnosis for men in the more affluent groups, with a higher proportion being referred for potentially curative resection.

## Figures and Tables

**Figure 1:**
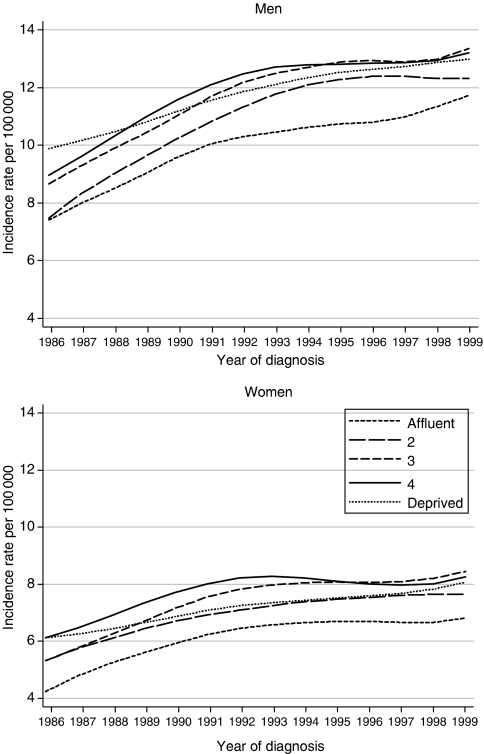
Trends in the age-standardised incidence of oesophageal cancer in adults aged 15–99 years, by sex and deprivation group: England and Wales, 1986–1999.

**Table 1:** Trends in relative survival (%) by sex, time since diagnosis and calendar period of diagnosis: England and Wales, adults (15–99 years) diagnosed during 1986–1999 and followed up to 2001

		**Calendar period of diagnosis^a^**						**Average change (%)**		**Prediction^c^ for patients**	
		**1986–1990**		**1991–1995**		**1996–1999**		**every 5 years^b^**		**diagnosed during 2000–2001**	
**Time since diagnosis**		**Survival (%)**	**95% CI**	**Survival (%)**	**95% CI**	**Survival (%)**	**95% CI**	**Survival (%)**	**95% CI**	**Survival (%)**	**95% CI**
1 year	Men	**23.8**	(23.1, 24.6)	**26.7**	(26.0, 27.5)	**29.6**	(28.7, 30.4)	3.7**	(2.1, 5.3)	**30.6**	(29.4, 31.7)
	Women	**24.4**	(23.4, 25.4)	**25.9**	(25.0, 26.8)	**26.8**	(25.8, 27.8)	**1.3**	(−0.6, 3.3)	**27.2**	(25.8, 28.6)
5 years	Men	**6.3**	(5.9, 6.9)	**6.3**	(5.8, 6.7)	**7.6**	(7.0, 8.2)	1.7**	(0.6, 2.8)	**8.0**	(7.2, 8.7)
	Women	**7.4**	(6.8, 8.1)	**7.4**	(6.9, 8.0)	**7.2**	(6.5, 8.0)	**−0.3**	(−1.7, 1.0)	**7.8**	(6.9, 8.8)
10 years	Men	**5.1**	(4.6, 5.6)	**4.8**	(4.3, 5.2)			2.0*	(0.2, 3.7)	**6.5**	(5.7, 7.3)
	Women	**6.2**	(5.6, 6.8)	**6.3**	(5.7, 6.9)			**0.7**	(−1.7, 3.1)	**6.5**	(5.6, 7.5)

CI=confidence interval.

a Survival estimated with cohort or complete approach (see Rachet *et al*, 2008).

b Mean absolute change (%) in survival every 5 years, adjusted for deprivation (see Rachet *et al*, 2008).

c Survival estimated with hybrid approach (see Rachet *et al*, 2008).

^*^P<0.05; ^**^P<0.01.

**Table 2:** Trends in the deprivation gap in relative survival (%) by sex, time since diagnosis and calendar period of diagnosis: England and Wales, adults (15–99 years) diagnosed during 1986–1999 and followed up to 2001

		**Calendar period of diagnosis^a^**						**Average change (%)**		**Prediction^c^ for patients**	
		**1986–1990**		**1991–1995**		**1996–1999**		**every 5 years^b^**		**diagnosed during 2000–2001**	
**Time since diagnosis**		**Deprivation gap (%)**	**95% CI**	**Deprivation gap (%)**	**95% CI**	**Deprivation gap (%)**	**95% CI**	**Deprivation gap (%)**	**95% CI**	**Deprivation gap (%)**	**95% CI**
1 year	Men	−3.3**	(−5.6, −1.0)	−5.2**	(−7.3, −3.0)	−4.8**	(−7.2, −2.4)	**−0.8**	(−2.6, 0.9)	−5.0**	(−8.4, −1.6)
	Women	**−2.0**	(−4.8, 0.9)	−4.1**	(−6.8, −1.4)	**−2.1**	(−5.0, 0.9)	**−0.1**	(−2.3, 2.0)	**−3.9**	(−8.0, 0.2)
5 years	Men	**0.7**	(−0.7, 2.1)	**−0.8**	(−2.0, 0.5)	−1.9*	(−3.8, −0.1)	−1.4*	(−2.6, −0.1)	−2.6*	(−4.8, −0.4)
	Women	**−0.9**	(−2.8, 1.0)	−1.8*	(−3.5, −0.1)	**−0.2**	(−2.4, 2.0)	**0.3**	(−1.2, 1.8)	**−0.3**	(−2.9, 2.3)
10 years	Men	1.5*	(0.1, 2.9)	**−1.2**	(−2.5, 0.2)			−2.7**	(−4.6, −0.7)	−2.3*	(−4.6, 0.0)
	Women	**−0.2**	(−2.1, 1.7)	**−1.0**	(−2.8, 0.9)			**−0.7**	(−3.4, 1.9)	**0.1**	(−2.5, 2.7)

CI=confidence interval.

a Survival estimated with cohort or complete approach (see Rachet *et al*, 2008).

b Mean absolute change (%) in the deprivation gap in survival every 5 years, adjusted for the underlying trend in survival (see Rachet *et al*, 2008).

c Survival estimated with hybrid approach (see Rachet *et al*, 2008).

^*^P<0.05; ^**^P<0.01.
